# Striving for a Higher Completion Rate of Patient Reported Outcome Measures in Kent and Medway NHS Trust

**DOI:** 10.1192/bjo.2024.426

**Published:** 2024-08-01

**Authors:** Leopold Rudolph, Isabel Barnett

**Affiliations:** 1 Oxleas NHS Foundation Trust, London, United Kingdom; 2 Maidstone & Tunbridge Wells NHS Trust, Kent, United Kingdom

## Abstract

**Aims:**

A Patient Reported Outcome Measure (PROM) is a form that patients complete about their health status at a single point in time. The Recovering Quality of Life (ReQoL) questionnaire is a new PROM, developed in partnership with mental health service users to enable them to report on their mental state, and can be utilised by clinicians to track progress. The Commissioning for Quality and Innovation (CQUIN) framework set a target that 40% of adult and older adult patients accessing secondary mental health services should have their PROM recorded at least twice in a 6-month period. The primary aim of this quality improvement project was for 50% of patients under the Kent and Medway NHS Trust (KMPT) to be ReQoL compliant.

**Methods:**

Following engagement with various stakeholders, a survey was circulated to better understand the barriers stopping staff from facilitating ReQoL completion. Moreover, a poster was created to raise awareness of ReQoLs and illustrate the practicalities behind gathering and recording patient scores. Additionally, local ‘champions’ were assigned for each community/inpatient mental health team to foster a sense of responsibility for PROM collection. Data on PROM compliance was obtained monthly, with meetings subsequently organised to scrutinise the results and brainstorm further ideas to drive improvement, such as providing patients with paper ReQoL copies to fill out in advance of their consultation/ward round.

**Results:**

The survey revealed that 23% of staff were unfamiliar with the ReQoL questionnaire, and only 31% routinely obtained and inputted ReQoLs. A lack of time to assist patients in filling out PROMs was the main barrier cited by staff, alongside ambiguity as to whose job it was to ensure ReQoL collection. Through the distribution of the poster, the establishment of local leads and other changes such as the paper ReQoL initiative, there was a notable uptick in the rates of PROM completion. Indeed, over a 4-month period, compliance rose locally from 46% to 61% at the acute inpatient unit, and from 0 to 21% in the community mental health service. However, over KMPT as a whole, change was modest.

**Conclusion:**

This was a successful quality improvement project, resulting in an increase in PROM completion rates, especially at a local level. The measures implemented, particularly the poster and formation of ReQoL leads, were effective – although more work and participation is required to change Trust-wide compliance. Future ideas include adding a ReQoL tool into nurse/doctor clerking templates to reduce friction in completing PROMs.

